# Head-to-Head Comparison of DH3 HPV Test and HC2 Assay for Detection of High-Risk HPV Infection in Residual Cytology Samples from Cervical Cancer Screening Setting: Baseline and 3-Year Longitudinal Data

**DOI:** 10.1128/spectrum.01570-21

**Published:** 2022-02-16

**Authors:** Yunfeng Fu, Xiao Li, Ying Li, Weiguo Lu, Xing Xie, Xinyu Wang

**Affiliations:** a Centre for Diagnosis & Treatment of Cervical Diseases, Women’s Hospital, School of Medicine, Zhejiang University, Hangzhou, China; b Department of Gynecologic Oncology, Women’s Hospital, School of Medicine, Zhejiang University, Hangzhou, China; University of Arizona

**Keywords:** cervical cancer screening, cervical intraepithelial neoplasia, human papillomavirus, Hybrid Capture 2, DH3 HPV

## Abstract

The authors compared the clinical performance of DH3 human papillomavirus (HPV) assay, which detects 14 high-risk HPVs with 16/18 genotyping based on hybrid capture technique, and Hybrid Capture 2 (HC2) test for women undergoing cervical cancer screening. A total of 7, 263 residual cytology specimens from an adjudicated cohort with 3-year follow-up were tested by the DH3 assay and the HC2 test. Assay results were compared with each other and to histology review. The overall agreement between the DH3 assay and the HC2 test was 99.2% (κ = 0.938). At baseline, DH3 had the equal sensitivity to that of HC2 for cervical intraepithelial neoplasia (CIN) grade 2 or higher (CIN2+, *n* = 75) and CIN grade 3 or higher (CIN3+, *n* = 45), 98.67% and 97.78%, respectively. After 3 years of follow-up, the sensitivity for CIN2+ (*n* = 133) and CIN3+ (*n* = 74) were both similar between DH3 and HC2 (95.49% vs 94.74%, 95.95% vs 95.95%, respectively, all *P* > 0.05). The respective specificity for CIN2+ or CIN3+ did not differ between the two tests. A noninferiority test showed that both sensitivity and specificity of DH3 for CIN2+ and CIN3+ were noninferior to those of HC2 at baseline and after 3-year follow-up, respectively (all *P* < 0.001). When used in primary screening strategy, the DH3 assay would yield an immediate sensitivity of 92% for CIN2+. DH3 HPV performs equally to HC2 for the detection of high-grade lesions in cervical cancer screening and has a potential advantage in primary screening strategy due to HPV16/18 genotyping.

**IMPORTANCE** The benefits of testing for high-risk human papillomavirus (hrHPV) in cervical cancer screening have already been demonstrated. Hybrid Capture 2 (HC2) is the best validated HPV assay and has been considered the gold standard for hrHPV testing. However, HC2 cannot discriminate HPV16 and 18 from the other hrHPV types, which greatly limited the application of HC2 in cervical cancer screening. The DH3 human papillomavirus (HPV) is a recently developed assay based on hybrid capture technique like to HC2, which can specifically identify HPV 16/18 on the basis of detecting the 13 hrHPV types targeted by HC2 as well as HPV66. This comparative study of the two assays for detection of hrHPV infection in residual cytology samples from cervical cancer screening setting reveals that DH3 HPV provides a perfect alternative to HC2 in detecting hrHPV infection and identifying cervical precancer, while allowing concurrent HPV 16/18 genotyping.

## INTRODUCTION

Cervical cancer is the fourth most common cancer in women worldwide, with over 500,000 new cases annually, most of which occur in developing countries (1). Almost all cervical cancer and its precursors are caused by persistent high-risk human papillomavirus (hrHPV) infection (2). Over the past 2 decades, hrHPV testing has gradually been proven as an effective strategy for the prevention and early detection of cervical cancer (3, 4). In 2012, the International Agency for Research on Cancer (IARC) classified 12 hrHPV types (HPV16, HPV18, HPV31, HPV33, HPV35, HPV39, HPV45, HPV51, HPV52, HPV56, HPV58 and HPV59) as 1A carcinogens (being carcinogenic) (5). Whereas HPV68 was considered as a 2A carcinogen (probably carcinogenic) (5). These 13 hrHPV types have been linked to 96% of cervical cancers, with HPV16 and HPV18 together being responsible for approximately 70 % of cases (6).

Hybrid Capture 2 (HC2; Qiagen, Germany) hrHPV DNA test, the first hrHPV test approved by the U.S. Food and Drug Administration (FDA), was designed to detect these 13 hrHPV types. HC2 assay is based on hybrid capture technique without PCR, which can reduce the risk of false-positive caused by cross-contamination. Detection of full-length human papillomavirus (HPV) DNA is another advantage of HC2, which can reduce to miss HPV that has been disrupted due to viral integration or point mutations (7). In fact, HC2 is the best clinically validated HPV assay and has been considered the reference standard for hrHPV testing (8). However, HC2 assay cannot discriminate HPV16 and 18 from the other hrHPV types. This disadvantage greatly limited the application of HC2 in cervical cancer screening. Currently, primary HPV testing with HPV16/18 genotyping has been recommended for cervical cancer screening by several important guidelines (9, 10).

The DH3 (Dalton, China) hrHPV DNA test is a recently developed assay based on hybrid capture technique like to HC2 (11, 12). It can detect 13 hrHPV types targeted by the HC2 test as well as HPV66, which is classified as a 2B carcinogen (possibly carcinogenic) by IARC (5). In addition, this non-PCR based assay can specifically identify HPV 16/18 without the need for a separate test. Recently, our cross-sectional study found that DH3 HPV performed similarly to Cobas 4800 HPV, a PCR-based assay with concurrent genotyping for HPV 16 and 18, in primary screening strategy for women aged 25–65 years (11). However, concordance study comparing to HC2 was more important because it was considered the gold standard. In theory, design similarity between the two assays will result in high levels of agreement. Nevertheless, it is urgent to know whether the modified designs of DH3 HPV (adding detection of HPV 66 with concurrent HPV16/18 genotyping) affect the concordance in clinical practices.

In this study we compared the concordance of DH3 HPV to the HC2 test using residual cytology samples from cervical cancer screening setting. In addition, we present here the first longitudinal data of 3 years follow-up regarding the clinical performance for the detection of high-grade cervical intraepithelial neoplasia (CIN) by the DH3 assay in comparison to the HC2 test.

## RESULTS

### Agreement between DH3 and HC2 assay.

The mean age of the 7,263 women was 47.2 years (range, 21–71 years), with 63.6% aged 45 years or older. At baseline, of the 7263 samples, 691 (9.51%) were DH3 HPV-positive, including 152 (2.09%) DH3 HPV16/18-positive, whereas 663 (9.12%) were HC2 HPV-positive. In total, 43 samples were DH3 HPV-positive but HC2 HPV-negative, 15 samples were DH3 HPV-negative but HC2 HPV-positive. The overall agreement between the DH3 HPV assay and the HC2 test was 99.20%, the positive agreement was 97.74%, and the negative agreement was 99.35% (Table 1). The kappa coefficient for the overall agreement was 0.938, indicating almost perfect agreement.

**TABLE 1:** Agreement between the DH3 HPV assay and the HC2 test^*a*^

Assay	Result	HC2		Total (%)
		Positive	Negative	
DH3	Positive	648	43	691 (9.51)
	Negative	15	6557	6572 (90.5)
Total (%)		663 (9.12)	6600 (90.9)	7263 (100)

a Values are number of specimens. Overall agreement: 99.20% (7205/7263). Positive agreement: 97.74% (648/663). Negative agreement: 99.35% (6557/6600). Kappa coefficient: 0.938 (95% CI, 0.920-0.956).

### Clinical performance for disease detection.

Cervical disease status of this cohort and the corresponding HPV results at baseline are shown in Table 2. A total of 407 women had a verified histology result at baseline. Among them, one case of cervical cancer, 44 cases of CIN3, 30 cases of CIN2, and 82 cases of CIN1 were identified. Eventually, 5840 women completed the 3-year follow-up. In addition to the 75 women with CIN2+ identified at baseline, 29 (0.5%) women had CIN2 and 29 (0.5%) women had CIN3+ identified during follow-up.

**TABLE 2:** Disease status at baseline and after 3 years of follow-up and the corresponding DH3 and HC2 results at baseline

Disease status	Participants, N (%)	DH3+			DH3−	
		HC2+	HC2−		HC2+	HC2−
Disease status at baseline						
Total no.	7,263	648	43		15	6,557
Normal^*a*^	7,106 (97.84)	498	43		14	6,551
CIN1	82 (1.13)	76	0		1	5
CIN2	30 (0.41)	30	0		0	0
CIN3	44 (0.61)	43	0		0	1
Cancer	1 (0.01)	1	0		0	0
CIN2+	75 (1.03)	74	0		0	1
CIN3+	45 (0.62)	44	0		0	1
Disease status after 3-yr follow-up						
Total no.	5,840^*b*^	558	25		0	5,257
Normal^*a*^	5,587 (95.67)	346	27		0	5,214
CIN1	120 (2.05)	83	0		3	34
CIN2	59 (1.01)	55	1		0	3
CIN3	71 (1.22)	69	0		0	2
AIS	1 (0.02)	0	0		0	1
Cancer	2 (0.03)	2	0		0	0
CIN2+	133 (2.28)	126	1		0	6
CIN3+	74 (1.27)	71	0		0	3

*^a^*Including unverified women without indications of colposcopy or biopsy.

*^b^*1423 women without CIN2+ at baseline were excluded from the final analysis because of lost follow-up.

The performance of DH3 HPV and HC2 test for identifying CIN2+ or CIN3+ at baseline and over 3-year follow-up are presented in Table 3. At baseline, the DH3 assay had the equal sensitivity to that of the HC2 test for the detection of CIN2+ and CIN3+, 98.67% (95% CI, 91.79%-99.93%) and 97.78% (95% CI, 86.77%-99.88%), respectively. The difference in specificity for CIN2+ and CIN3+ was not statistically significant between the DH3 assay and the HC2 test (*P* = 0.416 and *P* = 0.427, respectively). A noninferiority test revealed that both the clinical sensitivity (U = 32.5, *P* < 0.001) and specificity (U = 179.8, *P* < 0.001) of DH3 HPV for the detection of CIN2+ were noninferior to those of HC2 at baseline. Likewise, we found that both the clinical sensitivity (U = 26.6, *P* < 0.001) and specificity (U = 170.4, *P* < 0.001) of DH3 HPV for the detection of CIN3+ were noninferior to those of HC2 at baseline.

**TABLE 3:** The efficacy of DH3 HPV and HC2 test for identifying CIN2+ or CIN3+ at baseline and over 3-year follow-up

Status	Assay	Sensitivity			Specificity			NPV			PPV	
		%	95% CI		%	95% CI		%	95% CI		%	95% CI
Baseline												
CIN2+ (*n* = 75)	HC2 HPV	98.67	91.79–99.93		91.81	91.14–92.42		99.98	99.90–100		11.16	8.92–13.87
	DH3 HPV	98.67	91.79–99.93		91.43	90.75–92.06		99.98	99.90–100		10.72	8.56–13.33
	DH3 HPV16/18	57.33	45.40–68.51		98.48	98.17–98.75		99.55	99.4–99.7		28.29	21.44–36.26
	Cytology	76.00	64.50–84.79		97.63	97.25–97.97		99.74	99.59–99.84		25.11	19.71–31.37
CIN3+ (*n* = 45)	HC2 HPV	97.78	86.77–99.88		91.42	90.75–92.05		99.98	99.90–100		6.64	4.92–8.88
	DH3 HPV	97.78	86.77–99.88		91.05	90.36–91.69		99.98	99.90–100		6.38	4.72–8.53
	DH3 HPV16/18	57.78	42.24–72.01		98.25	97.92–98.54		99.73	99.6–99.8		17.11	11.67–24.25
	Cytology	77.78	62.52–88.29		97.34	96.94–97.69		99.86	99.86–99.93		15.42	11.11–20.93
Over 3-yr												
CIN2+ (*n* = 133)	HC2 HPV	94.74	89.06–97.67		92.43	91.71–93.10		99.87	99.71–99.99		22.58	19.22–26.32
	DH3 HPV	95.49	90.02–98.15		92.01	91.28–92.69		99.89	99.74–99.95		21.78	18.54–25.40
	DH3 HPV16/18	44.36	35.84–53.22		98.70	98.37–98.97		98.70	98.37–98.98		44.36	35.84–53.22
	Cytology	48.87	40.16–57.65		97.69	97.25–98.05		98.79	98.47–99.06		32.99	26.57–40.09
CIN3+ (*n* = 74)	HC2 HPV	95.95	87.82–98.95		91.55	90.80–92.25		99.94	99.82–99.99		12.72	10.13–15.84
	DH3 HPV	95.95	87.82–98.95		91.12	90.35–91.84		99.94	99.82–99.99		12.18	9.69–15.18
	DH3 HPV16/18	45.95	34.44–57.87		98.28	97.90–98.60		99.30	99.03–99.49		25.56	18.58–33.99
	Cytology	52.70	40.83–64.29		97.26	96.80–97.66		99.38	99.13–99.56		19.80	14.61–26.19

After 3 years of follow-up, the sensitivity for detection of CIN2+ and CIN3+ were both similar between DH3 and HC2 (*P* = 0.776 and *P* = 1.0, respectively). The specificity for detection of CIN2+ and CIN3+ were also similar between the two HPV assays (*P* = 0.402 and *P* = 0.408, respectively). A noninferiority test showed that both the clinical sensitivity and specificity of DH3 HPV for detection of CIN2+ (U = 47.4, *P* < 0.001; U = 189.3, *P* < 0.001) and CIN3+ (U = 39.4, *P* < 0.001; U = 189.6, *P* < 0.001) were also noninferior to those of HC2 over 3-year follow-up, respectively.

### Comparison of DH3 HPV and HC2 in primary screening strategy.

In Table 4, we present the results of the performance of DH3 HPV and HC2 in primary screening strategy. For HC2 primary screening, triaging HC2-positive women using LBC would yield a PPV for immediate colposcopy of 32.37%, with an immediate sensitivity for CIN2+ of 74.67%. For DH3 HPV primary screening, triaging DH3-positive women using the concurrent HPV16/18 genotyping and/or LBC and referring women who tested positive in either triage test would increase the immediate sensitivity to 92.0%, with a PPV of 36.9%. Compared with HC2 primary screening, only slightly more women would be referred in DH3 primary screening (187 versus 173) but 23.2% of additional CIN2+ cases would be identified immediately.

**TABLE 4:** Sensitivity and PPV for CIN2+ of HC2 and DH3 HPV primary screening at baseline

Screening strategy	No. referred to colposcopy	No. of CIN2+ found immediately	Referral/case ratio	Immediate sensitivity for CIN2+ (% [95% CI])	PPV of referral for CIN2+ (% [95% CI])
HC2 primary screening	173	56	3.09	74.67 (63.08–83.69)	32.37 (25.58–39.96)
					
DH3 primary screening	187	69	2.71	92.0 (82.79–96.71)	36.9 (30.06–44.29)

## DISCUSSION

HC2 is the most frequently used hrHPV assay worldwide. It was the data of HC2 assay from the longitudinal cohort of more than 1.5 million patients at Kaiser Permanente Northern California that became the cornerstone of 2019 ASCCP guidelines (13, 14). Similar to HC2, DH3 HPV is also based on the well-designed hybrid capture technology, which can be easily performed in general laboratories. Compared with HC2, concurrent HPV16/18 genotyping and additional detection of HPV 66 were the two technical modifications of DH3. As a possibly carcinogen classified by IARC, HPV 66 is a target genotype of many hrHPV assays, including Cobas 4800, Cervista, Aptima, and Onclarity HPV (8).

In this study, we compared DH3 HPV and the HC2 assay in residual LBC samples from an adjudicated cohort of 7263 women. We found that DH3 HPV had similar but slightly higher HPV-positive rates than HC2 HPV (9.51% versus 9.12%). There were only 58 (0.8%) discrepant specimens between the two assays, 43 of which were DH3 HPV-positive but HC2 HPV-negative. This is not unexpected, because the additional HPV 66 is covered by DH3 assay. A retrospective study showed that the prevalence of HPV-66 was approximately 0.94% among women attending cervical cancer screening in the same province of China (15).

As expected, the analytical concordance was quite good between the DH3 assay and the HC2 test. We found that the overall agreement was 99.20% between the two assays, the positive agreement was 97.74%, and the negative agreement was 99.35%. The kappa coefficient for the overall agreement was 0.938, indicating almost perfect interrater agreement. These results showed that specifically identifying HPV16/18 on the hybrid capture platform did not affect the overall efficacy of hrHPV detection. DH3 HPV provides a perfect alternative to HC2 in detecting hrHPV infection, while allowing concurrent HPV 16/18 genotyping.

In addition to the assessment of analytical concordance, the most important consideration when evaluating a new assay for detection of hrHPV in cervical screening is the clinical performance. A clinically useful hrHPV assay should have balanced the sensitivity and specificity for CIN2+ to ensure reliable detection of women with high-grade lesions and to minimize HPV-positive results in those without disease. It has been recommended that the candidate assay should have a clinical sensitivity and specificity for CIN2+ of not less than 90% and 98% of those of HC2, respectively (16).

In this study, of 7,263 women, 75 CIN2+ and 45 CIN3+ were identified at baseline. Both the clinical sensitivity and specificity of DH3 HPV for detection of CIN2+ and CIN3+ were noninferior to those of HC2, respectively. Furthermore, the sensitivity of DH3 HPV in identifying CIN2+ and CIN3+ was equal to that of HC2, 98.67% and 97.78%, respectively. The favorable clinical performance of DH3 was also confirmed in 3-year follow-up study, which comprised 5840 women with 133 CIN2+ and 74 CIN3+. These results indicate that the clinical value of DH3 HPV in identifying high-grade CIN is very similar to that of HC2.

There is growing evidence that HPV-based screening is more cost-effective than cytology or co-testing (4). Because HPV primary screening was recommended by EUROGIN, many developed countries were switching to hrHPV testing alone for cervical cancer screening (17). More recently, the American Cancer Society recommended primary HPV testing at a 5-year interval as the preferred screening strategy for all individuals being screened (18). However, an important consideration in HPV primary screening is the management of HPV-positive women. Reflex cytology is currently the only well validated triage test, which depends on the quality of cytology (4). Generally, HPV-positive women who have normal cytology should be retested in 1 year. This option might be less optimal because a small fraction of them might develop invasive cancer during the follow-up interval, especially in area without sufficient good cytologists. Furthermore, some HPV-positive women with a negative cytology will be lost to follow-up. Thus, additional stratification of HPV-positive women with normal cytology should be used to identify those at greater risk for high-grade lesions who warrant immediate referral. The ATHENA study demonstrated that genotyping for HPV16, HPV18, or both was a useful triage technique (19). In fact, Cobas 4800 and Onclarity HPV which both can identify HPV16/18 are currently the only two FDA-approved HPV tests for primary screening (18).

Considering the ability of concurrent HPV16/18 genotyping of DH3 assay, the potential value of DH3 HPV in primary cervical cancer screening was investigated in the present study. For this cohort, primary screening strategy with triaging HC2-positive women using reflex cytology would yield an immediate sensitivity for CIN2+ of only 74.67%. These results suggest that about 1/4 of high-grade lesions would be missed if HC2 primary screening was performed in areas with medium quality of cytology. If DH3 HPV primary screening was carried out with triaging DH3-positive women using the synchronous HPV16/18 genotyping and/or reflex cytology, the immediate sensitivity would increase to 92.0%. It should be noted that only slightly more women would be referred in DH3 HPV primary screening but 23.2% of additional CIN2+ cases would be identified immediately. These results support that DH3 HPV has a potential advantage in primary cervical screening. Consistently, our cross-sectional study reported previously in a routine screening population suggested that DH3 HPV performed similarly to Cobas 4800 in primary screening strategy for women aged 25–65 years (11).

There are several limitations of this adjudicated cohort study. Firstly, the specimens were stored for up to 3 years before they were retested with the DH3 HPV and the HC2 assay. However, the values obtained for HPV prevalence in this study are in agreement with our previous study showing a DH3 HPV positive of 9.9% in a cross-sectional screening (11). Secondly, there might be some verification bias because not all the women underwent biopsy. Usually, biopsy specimens were not taken from patients who had normal colposcopy impression with normal cytology results. Moreover, women with a result of negative co-testing were deferred to 3-year follow-up, and some women infected with other hrHPV not producing a detectable cytological abnormality would be deferred to 1-year follow-up. These might result in a lower disease prevalence in this cohort and a higher sensitivity for both HPV assays compared in this study. However, cumulative histological results through 3-year follow-up were included in the ultimate analysis.

## CONCLUSIONS

In addition to the perfect analytical agreement, evaluation of DH3 HPV in cervical cancer screening setting with baseline and 3-year longitudinal data showed that the clinical performance of DH3 HPV is not inferior to that of HC2. A considerable benefit of DH3 HPV test is the concurrent HPV16/18 genotyping which could serve as a valuable additional tool in patient risk stratification and management. In fact, triaging DH3-positive women using its inherent HPV16/18 genotyping and/or LBC would yield an immediate sensitivity for CIN2+ of 92%, which was significantly higher than triaging HC2-positive women using LBC. In summary, our results demonstrate that the DH3 HPV performs equally as well as HC2 for the detection of CIN2+ lesions in cervical cancer screening setting. Moreover, DH3 HPV has a potential advantage in primary screening strategy due to HPV16/18 genotyping and can be considered as a primary cervical screening option. Further evidence for the applicability of the DH3 HPV test in primary screening needs to be investigated.

## MATERIALS AND METHODS

### Specimens and histological diagnosis.

For this study, all specimens were from the residual cell preservation solution (PreservCyt, ThinPrep, Hologic, USA) of two screening projects with 3-year follow-up in Zhejiang province, China. After cytological examinations were routinely performed, the 7263 residual specimens of liquid-based cytology (LBC) in PreservCyt solution at baseline were stored in one walk-in refrigerator with 4°C for up to 3 years prior to HPV testing with both a DH3 test and an HC2 assay.

According to the respective referral indications of the two projects, colposcopy was performed by the same team from our hospital. Patients with visible acetic-white iodine-negative lesions were subjected to biopsy. Patients with abnormal cytology results and no visible acetic-white iodine-negative lesions were subjected to endocervical curettage. Patients who had a normal cytology and showed no visible lesion during colposcopy were not subjected to biopsy and were considered “no lesion”. The histological diagnoses of cervical lesions were divided into normal, low-grade squamous intraepithelial lesion (LSIL/CIN1), high-grade squamous intraepithelial lesion (HSIL)/CIN2, HSIL/CIN3 (including adenocarcinoma *in situ*), and carcinoma. Due to the ethical considerations, almost all of the women with negative co-testing results were not referred to colposcopy and were all regarded as LSIL or less.

**FIG 1:**
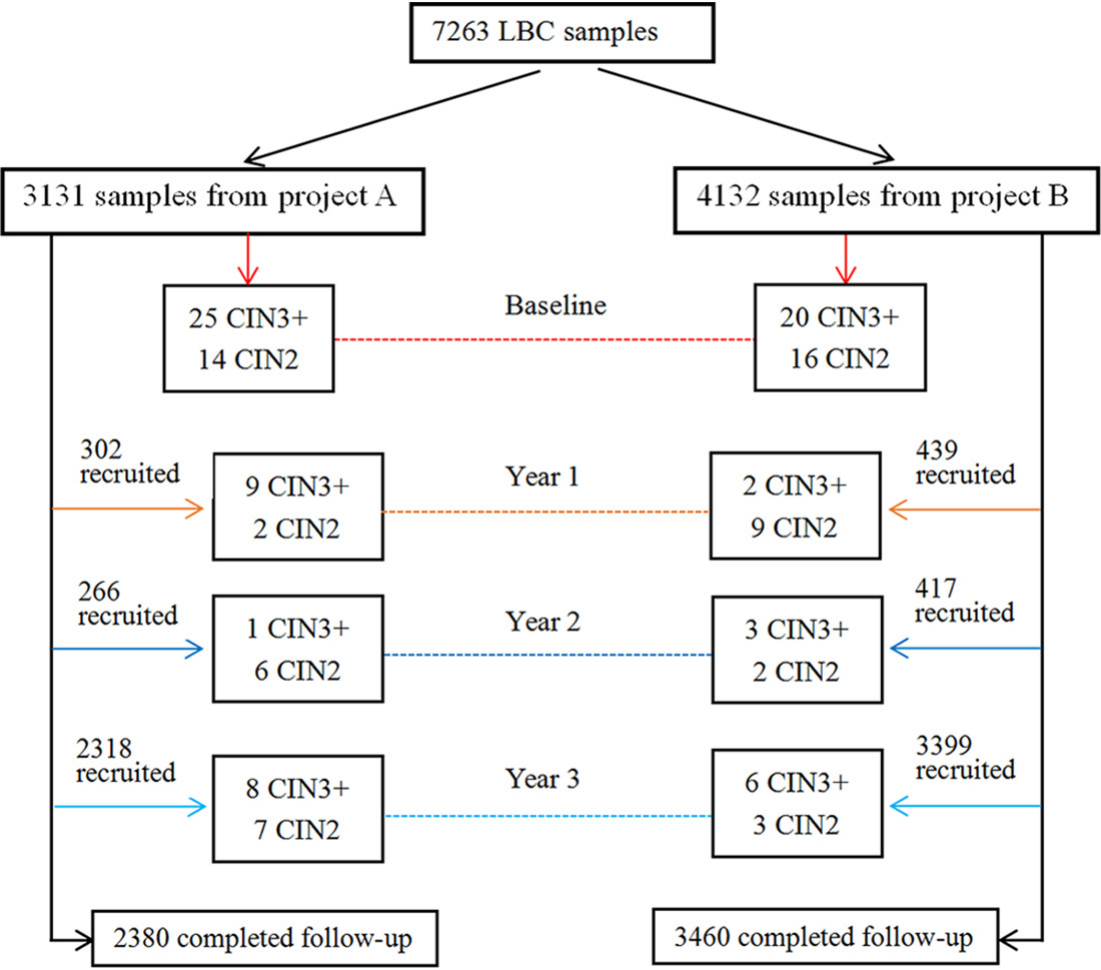
The study flow chart. LBC, liquid-based cytology; CIN, cervical intraepithelial neoplasia.

A summarized study flow chart is shown in Fig. 1. This study was performed in accordance with the 2013 Declaration of Helsinki and approved by the Ethics Committee of the Women's Hospital, School of Medicine, Zhejiang University (IRB-20200007-R).

### HPV test.

The residual LBC specimens were retested under blinded conditions with HC2 and DH3 HPV assay. HC2 HPV test was performed with an HC2 assay system according to the manufacturer’s protocol (Qiagen Inc.). DH3 HPV testing was performed as described previously (11). The results of DH3 HPV were divided as follows: HPV-, HPV16/18+ (result positive for genotype 16/18, with or without 12 other types), and HPV non-16/18+ (result negative for genotype 16/18 and positive for 1 or more of 12 other high-risk types).

### Statistical analysis.

The level of agreement between the two HPV tests was assessed by Cohen’s kappa statistics. Sensitivity, specificity, negative predictive value (NPV), and positive predictive value (PPV) were calculated by using the conventional contingency tables, and 95% confidence intervals (95% CI) were computed using Wilson score method. The chi-square test was used for intercomparison of proportions. Noninferiority of the clinical performance of DH3 HPV test versus HC2 assay was evaluated by noninferiority test for proportion (one-sided U test). The margins used for noninferiority were sensitivity for the detection of CIN2+ lesions of at least 90% and specificity for the detection of lesions less severe than CIN2 of at least 98% relative to the results of HC2, as previously described (16). Statistical analyses were carried out using SPSS (version 20.0) and SAS (version 9.1). The *P* values less than 0·05 were considered to indicate statistical significance.
